# Machine Learning-Based Measurement and Prediction of Ground Settlement Induced by Shield Tunneling Undercrossing Existing Tunnels in Composite Strata

**DOI:** 10.3390/s25051600

**Published:** 2025-03-05

**Authors:** Mei Dong, Mingzhe Guan, Kuihua Wang, Yeyao Wu, Yuhan Fu

**Affiliations:** Research Center of Coastal and Urban Geotechnical Engineering, Zhejiang University, Hangzhou 310058, China

**Keywords:** shield tunnel, monitoring, ground settlement, machine learning, prediction model, LSTM, BiLSTM

## Abstract

To address the issue of insufficient accuracy in traditional settlement prediction methods for shield tunneling undercrossing in composite strata in Hangzhou, this paper proposes a particle swarm optimization (PSO)-based Bidirectional Long Short-Term Memory neural network (Bi-LSTM) prediction model for high-precision dynamic prediction of ground settlement under small-sample conditions. Shield tunneling is a key method for urban tunnel construction. This paper presents the measurement and prediction of ground settlement caused by shield tunneling undercrossing existing tunnels in composite strata in Hangzhou. The longitudinal ground settlement curve resulting from shield tunnel excavation was analyzed using measured data, and the measured lateral ground settlement was compared with the Peck empirical formula. Using PSO, the performance of three machine learning models in predicting the maximum ground settlement at monitoring points was compared: Long Short-Term Memory neural network (LSTM), Gated Recurrent Unit neural network (GRU), and Bi-LSTM. The linear relationships between different input parameters and between input parameters and the output parameter were analyzed using the Pearson correlation coefficient. Based on this analysis, the model was optimized, and its prediction performance before and after optimization was compared. The results show that the Bi-LSTM model optimized with the PSO algorithm demonstrates superior performance, achieving both accuracy and stability.

## 1. Introduction

To address the growing traffic issues in China’s major cities, urban transportation systems have developed rapidly, and the scale and technological level of tunnel construction have reached new heights [1,2]. Shield tunneling is a key method for urban tunnel construction, offering advantages such as high safety standards, fast construction speed, small environmental disturbance, and limited impact on ground structures and the surrounding soil environment [3]. With the rapid development of urban tunnels in China, we are increasingly encountering situations where new tunnels cross various special strata [4]; when a new tunnel is excavated near existing structures, the resulting deformations can be quite complex [5,6]. For instance, the new tunnel crosses existing tunnels [7,8], pipelines [9], railways [10], and adjacent buildings [11]. Underground construction inevitably disturbs the surrounding soil layers and redistributes the original soil pressure. In recent years, frequent safety incidents caused by excessive ground settlement have made the prediction and control of settlement induced by shield tunneling a critical issue in tunnel construction [12].

Traditional methods for predicting ground settlement primarily include empirical solutions, theoretical analyses, and numerical simulations.

The empirical solution [13,14,15], represented by the Peck formula, offers a simple calculation and is convenient for engineering applications. However, it has poor geological adaptability, as the parameters for strata loss rate and settlement trough width must be derived from specific geological statistics. It also lacks a dynamic process, only predicting the final settlement form and failing to reflect real-time settlement evolution during shield tunneling. Additionally, it is difficult to consider the impact of shield construction parameters.

The theoretical solution [16,17,18] provides a clear physical model but relies on certain computational assumptions. By simplifying boundary conditions, it reduces infinite strata to a semi-infinite space, making it difficult to accurately reflect the interaction between shield excavation and the strata. It also struggles to account for the potential impacts of complex geology and shield construction methods on ground deformation. The calculation process is complex, making it unsuitable for real-time construction predictions and difficult to apply in practical engineering.

The numerical simulation method [19,20,21,22] can capture the intricate details of complex geology. For example, Vaghefi, M. [23] proposed a model for the interaction between underground structures and soils considering the Arbitrary Lagrangian–Eulerian (ALE) method using the Finite Element Method (FEM), which is relatively new. However, in general, the numerical simulation method faces difficulties in determining constitutive model parameters, and it comes with high modeling costs and time-consuming calculations, resulting in poor real-time performance. Additionally, the method lacks multi-field coupling, and current commercial software is unable to integrate the dynamic coupling process of grout permeation diffusion and soil stress fields.

The empirical solution offers a simple formula, making it easy to apply in engineering; however, it fails to account for the influence of shield construction parameters. The theoretical solution provides a clear physical model but is limited by certain assumptions, which hinder its ability to consider shield construction parameters. It also fails to accurately capture the interaction between shield excavation and strata, with a complex calculation process that is difficult to implement in practice [24]. The numerical simulation method, though useful, faces challenges in determining constitutive model parameters, with high modeling costs and time-consuming calculations [1].

In recent years, with the rapid advancement of computer and artificial intelligence technologies, machine learning has emerged as a key method for predicting ground settlement during shield tunneling. Machine learning algorithms exhibit high fault tolerance, adaptability, and strong nonlinearity. They handle multiparameter coupling effectively and generate predictions quickly, making them ideal for the timely and accurate acquisition of ground settlement data in real-world projects [12]. Compared with traditional methods, machine learning offers key advantages: (1) There are no constraints on the input parameter’s dimension. Shield construction parameters can be incorporated into the input data, and in theory, all relevant parameters can be included in the model. The training process automatically adjusts the weights of these parameters [25]. (2) Input parameters are typically derived from actual engineering data, making predictions directly applicable to the project. (3) Machine learning models demonstrate strong fitting ability.

Since Suwansawat and Einstein [26] first used the backpropagation neural network (BPNN) to establish a predictive relationship between shield tunnel characteristics and ground deformation, various machine learning algorithms have since emerged. The existing machine learning-based research methods for ground settlement caused by tunnel construction include the backpropagation neural network (BPNN) [27,28] and wavelet neural network (WNN) [29,30] within artificial neural networks (ANNs), as well as the support vector machine (SVM) [31,32] and random forest (RF) [33,34,35]. Machine learning models typically focus on three key components: input parameters, output parameters, and prediction algorithms. The shield tunneling parameters are part of the input parameters, while the output parameter is the maximum ground settlement or longitudinal ground settlement, which are essentially time series, where historical data influence current and future ground settlement. While the aforementioned machine learning methods are suitable for most regression tasks, their prediction performance is suboptimal when applied to time series data [11]. To address this issue, researchers have developed various sequence modeling methods, including recurrent neural network (RNN) [36,37], Long Short-Term Memory neural network (LSTM) [38,39,40], and Gated Recurrent Unit neural network (GRU) [41,42,43]. RNN models often encounter long-term dependency issues when processing time series, leading to problems such as gradient explosion or gradient vanishing. LSTM and GRU models can mitigate these issues to some extent.

However, several issues persist in current research: (1) Most current studies use the maximum settlement at the ground axis as the output parameter (prediction target), which is relatively simplistic. (2) Few studies have applied machine learning methods to predict ground settlement in shield tunneling under complex working conditions, and the effectiveness of these predictions remains uncertain. (3) In real-world conditions with small datasets, machine learning-based ground settlement prediction methods are prone to overfitting and often exhibit poor performance.

With the ongoing expansion of urban development in Hangzhou, subways have become a crucial component in driving the city’s growth. However, Hangzhou’s landscape is characterized by the widespread presence of Qiantang River alluvial silt layers, primarily composed of sandy silt, which represents a typical poor foundation in flatland areas [44,45]. The shield tunnel construction project studied in this paper faces a complex situation of poor foundation containing sandy silt, undercrossing existing tunnels, and a small dataset. The prediction capabilities of LSTM, GRU, and Bi-LSTM models for ground settlement are compared to select the most effective model. In addition, the linear relationship between different input parameters and between input parameters and the output parameter is revealed and analyzed through the Pearson correlation coefficient, and the model is optimized accordingly.

## 2. Monitoring and Analysis 

### 2.1. Project Overview

**Figure 1:**
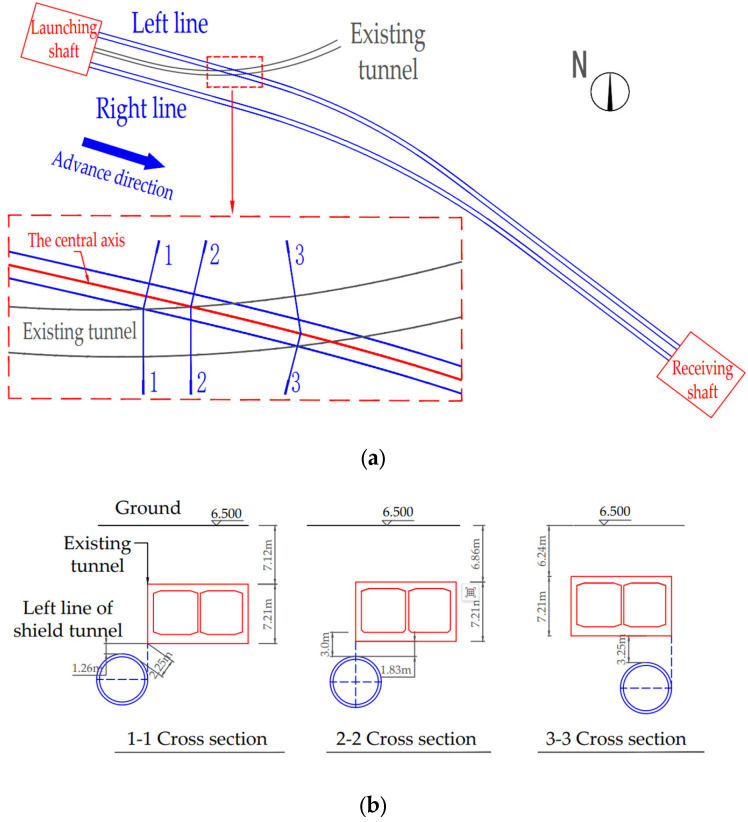
Position relationship between the left line of the new shield tunnel and the existing tunnel: (**a**) plan view of the position relationship between the new and existing tunnels; (**b**) vertical position relationship between the left line of the new shield tunnel and the existing tunnel.

The shield tunnel project discussed in this paper is located in Hangzhou, Zhejiang Province, China. It consists of two parallel tunnels constructed using the earth pressure balance (EPB) shield method. The left tunnel has a length of 848.691 m, with a burial depth ranging from 11.6 to 23.9 m, and a maximum slope of 27.9‰. After the interval section departs from the starting shaft, rings 123 to 180 cross under the existing tunnel. The existing tunnel was constructed using the open-cut method, and the two tunnels intersect at an oblique angle. Figure 1a shows the plan view of the relative positions of the new shield tunnel and the existing tunnel, while Figure 1b illustrates the vertical position relationship between the left tunnel of the new shield tunnel and the existing tunnel. The vertical distance between the new tunnel and the existing tunnel floor ranges from 1.26 m to 3.26 m.

### 2.2. Monitoring Scheme

#### 2.2.1. Layout of Monitoring Point

**Figure 2:**
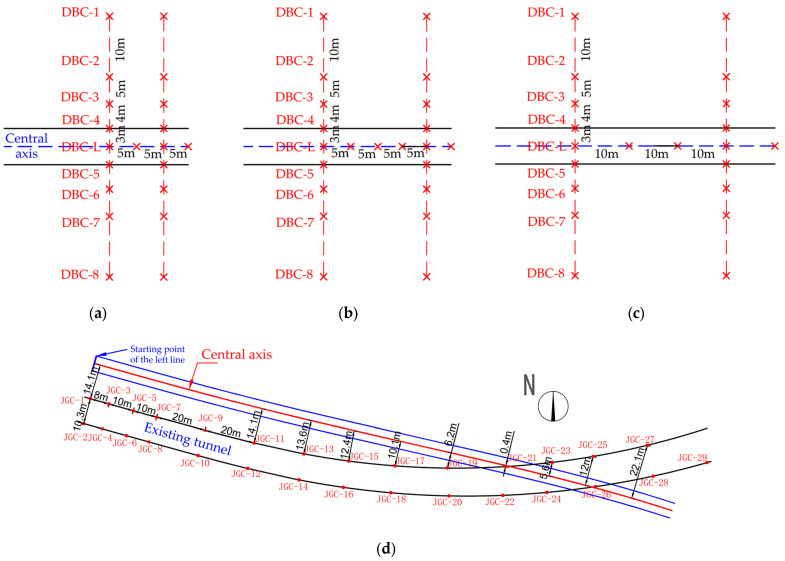
Layout of monitoring points: (**a**) layout of ground settlement monitoring points for rings 0–25 of the new tunnel; (**b**) layout of ground settlement monitoring points for rings 25–83 and 600–662 of the new tunnel; (**c**) layout of ground settlement monitoring points for rings 83–600 of the new tunnel; (**d**) layout of monitoring points in existing tunnels.

The ground settlement monitoring points for the new shield tunnel are arranged as shown in Figure 2a. For the area between the 0th and 25th rings (approximately 0–30 m) of the left tunnel line, monitoring points are placed every 5 m along the central tunnel axis, labeled as DBC-L. Additionally, a transverse monitoring cross-section is arranged every 10 m perpendicular to the tunnel axis, labeled as DBC1-DBC8. For the section between the 25th and 83rd rings, as well as between the 600th and 662nd rings (approximately 30–100 m and 723–798 m), monitoring points are arranged every 5 m along the tunnel axis, with lateral monitoring sections every 20 m perpendicular to the tunnel axis (Figure 2b). For the section between the 83rd and 600th rings (approximately 100–723 m), monitoring points are placed every 10 m along the tunnel axis, with lateral monitoring sections every 30 m perpendicular to the tunnel axis (Figure 2c). Furthermore, within the range from the 662nd to the 704th rings (approximately 798–848 m), monitoring points are set every 5 m along the tunnel axis, with lateral monitoring sections placed only at the 662nd and 704th rings. The transverse monitoring sections are symmetrically arranged along the tunnel axis, with measurement points located 3 m, 7 m, 12 m, and 22 m perpendicular to the tunnel axis.

The existing tunnel monitoring is shown in Figure 2d, with the excavation mileage of the new tunnel’s left line spanning from the 123rd to the 180th rings (approximately 152.8 m to 220.2 m), undercrossing the existing tunnel.

A total of 29 monitoring points are arranged in the existing tunnel, labeled as JGC, including No. 1, No. 3, No. 5, No. 7, and so on, with No. 27 being the left boundary monitoring point, while No. 2, No. 4, No. 6, and No. 8, as well as No. 26, No. 28, and No. 29 are the right boundary monitoring points. The distance between No. 1 and No. 3 on the left boundary is 8 m, while the distance between No. 3, No. 5, and No. 7 is 10 m. The distance between each monitoring point from No. 7 to No. 27 is 20 m. The distribution of monitoring points on the right boundary mirrors that on the left. After No. 11, the two tunnels gradually approach each other, reducing the distance between the monitoring points and the central axis of the left line of the new tunnel. After No. 19, the left line of the new tunnel passes under the existing tunnel. The No. 21 monitoring point is located only about 0.4 m from the central axis of the new tunnel’s left line, after which the two tunnels gradually diverge, and the distance between the monitoring points and the central axis increases.

#### 2.2.2. Method of Ground Settlement Monitoring

The ground settlement data in this study are derived from the high-precision leveling measurement network of the basic electronic leveling instrument (Leica DNA03, Leica Geosystems AG, Heerbrugg, Switzerland). The working principle involves using the scale’s barcode pattern as a reference signal stored within the instrument. During measurement, the built-in line decoder of the instrument captures the scale image within the instrument’s field of view as the measurement signal, which is then compared with the reference signal to obtain the line-of-sight height and horizontal distance [46]. The electronic leveling instrument is characterized by objective readings, fast measurement speed, modular recording, and ease of automated data processing [47].

The key technological advantages of Leica DNA03 in settlement monitoring during shield tunnel excavation are as follows:

(1) High-Precision Measurement: A pseudo-random binary-encoded indium wafer barcode scale is used, with a scale marking accuracy of ≤5 μm/m. After capturing the scale image via CCD, DNA03 utilizes a digital correlation algorithm (DCA) to compare the captured image with a pre-stored scale encoding template, thereby calculating the precise scale reading with a resolution of 0.01 mm.

(2) Deep Adaptation to Shield Tunnel Engineering Requirements: The instrument features a fast measurement mode that supports a BFFB (Back–Forward–Forward–Back) observation sequence, with a single-station observation time of ≤3 s, meeting the high-efficiency monitoring needs in tunnel construction areas. The GSI data format allows for direct recording of point numbers and height differences, seamlessly integrating with the adjustment software Leica GeoOffice v8.4, reducing manual entry errors.

(3) Spatial and Temporal Resolution Settings: The spatial resolution is as described in Section 2.2.1, and the temporal resolution involves conducting measurements once daily at 8:00 am during the shield tunneling phase.

#### 2.2.3. Sources and Range of Errors

The sources of errors are primarily composed of instrument error $u_{1}$, benchmark point stability $u_{2}$, and environmental interference $u_{3}$, with the following values:

(1) $$u_{1}=\frac{0.3}{\sqrt{2}}\times \sqrt{L}$$

(2) $$u_{2}=0.1$$

(3) $$u_{3}=0.1\times L$$

where 0.3 represents the nominal accuracy of Leica DNA03, which has a standard deviation of ±0.3 mm per kilometer of round-trip measurement. *L* is the distance between two adjacent working benchmark points, which is 0.15 km, and 0.1 represents the empirical environmental error value of ±0.1 mm/km. The calculated values are $u_{1}$ = 0.082 mm, $u_{3}$ = 0.015 mm, and, based on regulatory requirements, engineering needs, and actual conditions, $u_{2}$ = 0.1 mm.

Sensitivity analysis:

(4) $$u=\sqrt{u_{1}^{2}+u_{2}^{2}+u_{3}^{2}}$$

(5) $$i=\frac{u}{S_{avg}}$$

where u is the total error, which is calculated to be 0.13 mm. $S_{avg}$ is the average value of 126 monitoring point settlement data, which is 2.91 mm. The impact rate i is 4.4%, indicating that the error is small and falls within the acceptable range for engineering applications.

### 2.3. Longitudinal Ground Settlement

**Figure 3:**
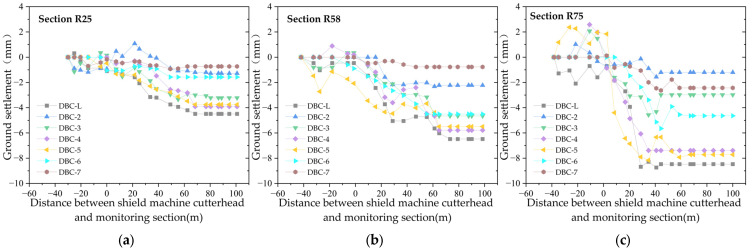
Longitudinal ground settlement curve before the left line of shield tunnel crosses under the existing tunnel: (**a**) section of the 25th ring; (**b**) section of the 58th ring; (**c**) section of the 75th ring.

**Figure 4:**
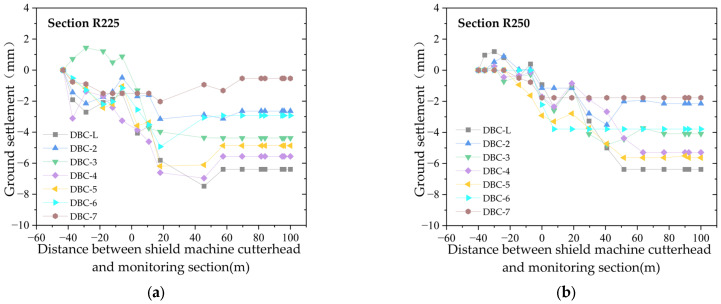
Longitudinal ground settlement curve after the left line of shield tunnel crosses under the existing tunnel: (**a**) section of the 225th ring; (**b**) section of the 250th ring.

The longitudinal settlement curves of the left line of the newly built shield tunnel are shown in Figure 3 and Figure 4. Figure 3 illustrates the longitudinal settlement curve of the left line before it crosses under the existing tunnel, including the 25th ring (approximately 30 m from the starting point of the left line), the 58th ring (approximately 70 m), and the 75th ring (approximately 90 m). Figure 4 shows the longitudinal settlement curve after it crosses, including the 225th ring (approximately 270 m) and the 250th ring (approximately 300 m). There are nine transverse monitoring points, namely DBC1–8 and DBC-L, with DBC1 and DBC8 being the two monitoring points farthest from the central axis of the left line of the tunnel (22 m). The measured data indicate that the ground settlement or uplift values at these two points are less than 0.5 mm, with more than half of the ground settlement values being 0.

Figure 3 and Figure 4 show that, except for the 25th monitoring section, which is about 30 m from the starting point of the left line, all the other monitoring points in the remaining sections begin monitoring once the shield machine cutterhead is 40–45 m away from the section, and continue until 100 m after the cutterhead passes through the section. Prior to the excavation face reaching the monitoring section, the ground experiences slight settlement or uplift, with a settlement range of approximately ±2 mm. After the excavation face reaches the monitoring section, the ground settlement increases, and tends to stabilize when the excavation face is 40 m to 60 m from the monitoring section. The final ground settlement is within 10 mm, with greater settlement values closer to the tunnel axis.

The final settlement values were normalized using the min–max normalization method, transforming the settlement data to the range [0, 1] according to the following equation:

(6) $$x_{i}=-\frac{x_{i}-x_{\min}}{x_{\max}-x_{\min}}$$

where $x_{i}$ is the original settlement data, and $x_{\min}$ is taken to be 0 because the settlement monitoring points DBC1 and DBC8, which are 22 m away from the center axis, are approximately equal to 0. $x_{\max}$ is the value of DBC-L at the axes of each monitoring section.

The Peck empirical formula [13,48] estimates the lateral ground settlement using the following equations:

(7) $$S(x)=S_{\max}\exp[-x^{2}/(2i^{2})]$$

**Figure 5:**
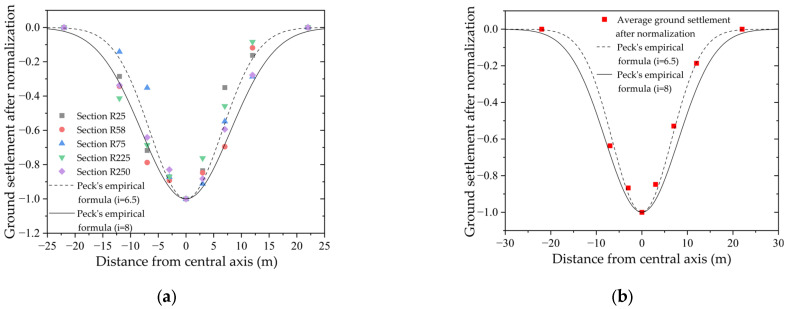
Normalized ground settlement: (**a**) normalized ground settlement values of each ring and the Peck empirical formula curve with i values of 6.5 and 8; (**b**) average normalized ground settlement and the Peck empirical formula curve with i values of 6.5 and 8.

(8) i=kh

where x is the horizontal distance from the tunnel axis, $S\left(x\right)$ is the ground settlement at position x, $S_{\max}$ is the maximum ground settlement above the tunnel axis, i is the ground settlement trough width coefficient, k is the ground settlement trough width parameter, h is the tunnel axis burial depth, and the value of i is 6.5 and 8 based on the analysis of the measured data from the Jiangnan section of the Qingchun Road Cross-River Shield Tunnel in Hangzhou by Wei Gang [49]. Figure 5a shows that the ground settlement value after normalization closely matches the ground settlement trough estimated by Peck’s empirical formula, and the ground settlement data are generally in line with Peck’s empirical formula. Figure 5b compares the average ground settlement value after minimum–maximum normalization with the Peck curve, and the results show that the average ground settlement value has a high degree of overlap with the Peck curve when i is taken as 6.5.

### 2.4. Settlement of Existing Tunnel

**Figure 6:**
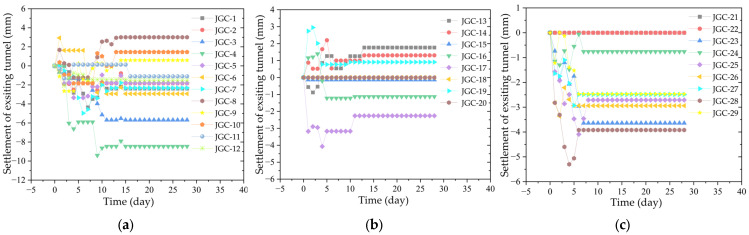
Settlement of existing tunnel: (**a**) longitudinal settlement curve of the existing tunnel in parallel section; (**b**) longitudinal settlement profile of the existing tunnel in the approach section; (**c**) longitudinal settlement curve of the existing tunnel in the undercrossing section.

The settlement monitoring point of the existing tunnel begins recording when the excavation face of the left line of the new tunnel reaches the monitoring point of the existing tunnel. Figure 6a shows the longitudinal settlement of the existing tunnel in the section where the existing tunnel and the left line of the new tunnel run nearly parallel. Figure 6b displays the longitudinal settlement of the existing tunnel in the section where the two tunnels are close to each other, and Figure 6c illustrates the longitudinal settlement of the existing tunnel in the undercrossing section. These figures indicate that the existing tunnel is affected by the excavation of the new shield tunnel, resulting in settlement. The settlement of the existing tunnel in the parallel section and the section near the shield tunnel tends to stabilize 10–15 days after the shield excavation face leaves, while the settlement in the undercrossing section stabilizes 6 days after the excavation face departs. This could be because the existing tunnel was preconditioned to accommodate the new shield tunnel. The retaining structure of the shield undercrossing section uses SMW piles, and when the existing tunnel was constructed, the undercrossing section of the shield tunnel was reinforced with mixing piles in advance. As a result, the strength, water-stopping capabilities, uniformity, and overall stability of the stratum were enhanced, leading to smaller settlement during shield excavation and rapid stabilization in the reinforced area [50].

## 3. Methodology

### 3.1. Machine Learning Networks

#### 3.1.1. Long Short-Term Memory Neural Networks (LSTM)

**Figure 7:**
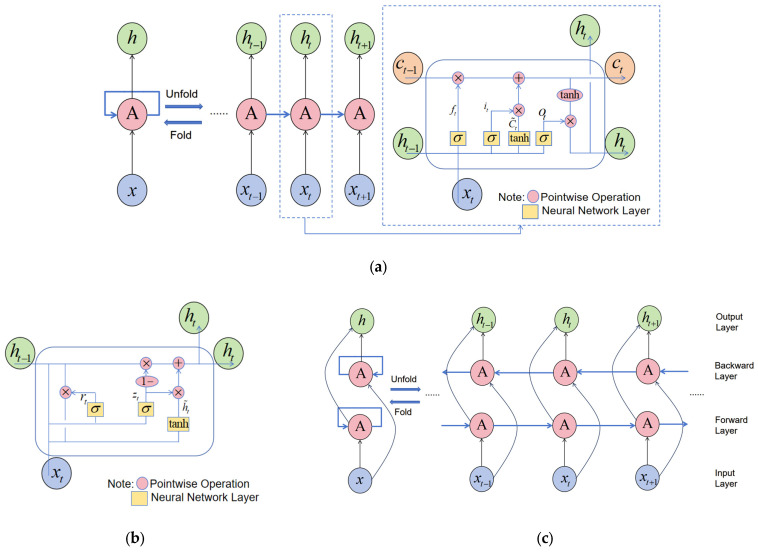
Deep learning networks: (**a**) LSTM model structure and its unit structure; (**b**) GRU unit structure; (**c**) Bi-LSTM model structure.

The LSTM (Long Short-Term Memory) neural network is a variant of the recurrent neural network (RNN). Traditional RNN models face the challenge of long-term dependency, which can lead to issues such as gradient vanishing and gradient explosion [51]. To address this, Sepp Hochreiter and Jürgen Schmidhuber [52] introduced the LSTM model in 1997. This model is capable of learning long-term dependencies and is particularly suited for processing and predicting time series data with significant intervals and delays between important events [53]. In an LSTM model, the calculation of a single neuron in the hidden layer involves two main components: cell state update and output value calculation. The neuron contains three gate functions: the forget gate, input gate, and output gate. These gates control the input value, memory value, and output value, determining which information and state from previous time steps should be retained and which should be discarded. This structure effectively preserves information over long periods. Figure 7a illustrates the LSTM model structure and its unit structure.

In Figure 7a, time is represented as t, $x_{t}$ is the input data of the LSTM unit, $h_{t-1}$ is the output of the LSTM unit at the previous time step, $c_{t}$ is the cell state at moment t, and $h_{t}$ is the output of the LSTM unit.

#### 3.1.2. Gated Recurrent Unit Neural Network (GRU)

GRU is a variant of LSTM proposed by Cho, K. et al. [54]. Its primary difference lies in the combination of the forget and input gates into a single update gate. This model controls the input, memory, and output values of the cell via the update and reset gates, effectively merging the cell state and hidden state. As a result, the GRU model is simpler than the LSTM model. The structure of the GRU model is analogous to that of the LSTM model shown in Figure 7a, with its unit structure depicted in Figure 7b.

#### 3.1.3. Bidirectional Long Short-Term Memory Neural Network (Bi-LSTM)

The LSTM model utilizes previous information to predict future information. In time series prediction, considering both the past and future information surrounding the prediction point can enhance prediction accuracy [55]. The Bi-LSTM neural network consists of two LSTM networks with opposite information flow directions. The two-layer structure processes the time series data together, and their output results are integrated in the output layer for a more comprehensive analysis of data features and patterns. Its structure is shown in Figure 7c. The lower layer is the forward layer, while the upper layer is the backward layer. The unit structure of each layer is identical to the LSTM unit structure shown in Figure 7a.

### 3.2. Particle Swarm Optimization (PSO)

The selection of model hyperparameters in machine learning significantly affects the performance of prediction models. Therefore, it is essential to systematically combine and select the various hyperparameters to ensure that the prediction model achieves its maximum performance. Currently, the optimal combination of hyperparameters can be determined through methods such as manual trial and error, grid search, random search, and optimization algorithms [38].

Particle swarm optimization (PSO) is an optimization algorithm designed to find the optimal solution for a model. It is characterized by fast convergence, high accuracy, and a low likelihood of getting stuck in local optima [56]. Each particle’s position corresponds to the value of an optimization parameter, and its fitness is used to evaluate the performance of that value in the model. The goal of the PSO algorithm is to optimize the parameters through multiple iterations, thereby maximizing the model’s prediction performance. Each particle computes its fitness based on the fitness function and continuously updates its velocity and position. The PSO algorithm continues to iterate until the optimal solution is found.

## 4. Prediction Model Parameter Selection

The data used in this paper were selected from the left line of the newly built shield tunnel, specifically from rings 0 to 250 (approximately 0 to 300 m). A total of 126 datasets were collected, including both input and output data. The input parameters include all factors that may cause ground settlement or heaving, typically including geological parameters, geometric parameters, and operational parameters [1,6]. The output parameter is the maximum ground settlement at the monitoring points.

### 4.1. Geological Parameter

**Figure 8:**
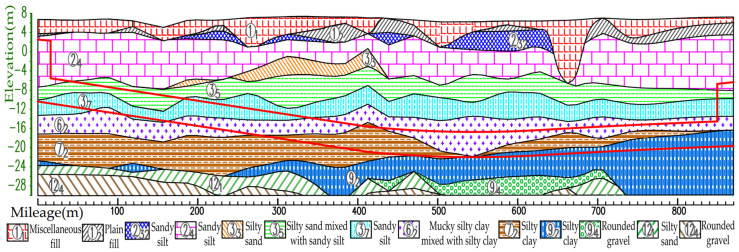
Geological profile of the left line of the shield tunnel.

The geological profile of the selected left line section of the shield tunnel is shown in Figure 8. The left line shield mainly traverses sandy silt, silty sand mixed with sandy silt, mucky silty clay mixed with silty clay, and silty clay. Table 1 presents the physical and mechanical parameters of the relevant soil layer, and the parentheses indicate the empirical value. As shown in the table, the physical and mechanical properties of each soil layer are quite different.

Chen. R.-P. et al. [34] proposed a new method to use different in situ test results as measurement factors according to the stratum type. By defining the thickness correction factor and the depth correction factor, the strength parameters of each stratum covered by the tunnel are corrected and weighted, so as to characterize the comprehensive stratum conditions of the tunnel. The formula is as follows:

(9) $$N^{\prime }=\sum_{i=1}^{n}\frac{t_{i}}{h}\cdot \frac{h_{i}}{h}\cdot N$$

where N is the standard penetration test blow number, $N^{\prime }$ is the modified standard penetration test blow number, $t_{i}$ is the thickness of the i-th soil layer, and $h_{i}$ is the depth of the i-th layer. $\frac{t_{i}}{h}$ represents the thickness index, $\frac{h_{i}}{h}$ represents the depth index, and the product of the two is the modified index. In addition to the modified standard penetration test blow number as an input parameter, the elastic modulus and static side pressure coefficient of the top and bottom strata of the existing tunnel and the new tunnel [25,39] are used as input parameters, and the geological parameters are shown in Table 2.

### 4.2. Geometric Parameter

In shield tunneling projects, the geometric parameters generally believed to affect ground settlement include the shield machine diameter, the shield tail gap, and tunnel depth [25]. Among them, shield machine diameter and shield tail gap are constants and have no effect on the prediction model results. Only tunnel depth is used as an input parameter. Additionally, due to the small dataset in this study, with only 126 samples, the data are extremely limited. The maximum ground settlement at the tunnel axis accounts for less than one-third of the total dataset. If the maximum ground settlement at the tunnel axis were used solely as the output for the model, it would likely lead to poor model fitting or overfitting. Therefore, the maximum ground settlement recorded at all monitoring points within the monitoring range is used as the output. As indicated in the longitudinal ground settlement analysis in Section 2.3, the distance between the monitoring point and the tunnel axis significantly affects the settlement value at the monitoring point. Consequently, the distance between the monitoring point and the tunnel axis must be considered as one of the geometric parameters.

This paper investigates the scenario in which a new shield tunnel passes under an existing tunnel in the engineering section. Previous studies have shown that the existing tunnel, located along the axis of the new tunnel, has a certain impact on ground settlement [57]. Therefore, the positional relationship parameters between the existing and new tunnels must be included in the input parameters. These positional relationship parameters include both horizontal and vertical distance parameters. The horizontal distance parameter refers to the horizontal distance between the midpoint of the excavation surface of the new tunnel and the centerline of the existing tunnel. The vertical distance parameter refers to the vertical distance between the midpoint of the excavation surface of the new tunnel and the centerline of the existing tunnel.

The tunnel depth and vertical distance parameters mentioned above are related to composite strata with uneven physical and mechanical properties. In order to reduce the adverse effects of composite strata on the prediction model, this paper introduces the equivalent layered method [58], which transform the uneven composite strata into uniform strata with the consistent mechanical properties. The calculation formula is as follows:

(10) $$h_{2}^{\prime }=h_{2}{\left(\frac{E_{2}}{E_{1}}\right)}^{a}$$

In the formula, $E_{1}$ represents the elastic modulus of the upper soil layer, $E_{2}$ and $h_{2}$ represent the elastic modulus and thickness of the lower soil layer, and a is the layer index. According to the research on the construction of new tunnels under existing tunnels, a is taken as 0.5 [59]. According to the equivalent layered method, the lower soil can be equivalent to an equivalent soil layer with the same physical and mechanical properties as the upper soil layer, and ${h^{\prime }}_{2}$ is the equivalent thickness of the soil layer. The geometric parameters are shown in Table 3.

### 4.3. Operational Parameter

A large number of parameters are recorded during shield tunneling, including thrust, cutterhead rotational torque, cutterhead rotational speed, excavation rate, screw conveyor speed, screw machine torque, penetration rate, chamber earth pressure, propulsion oil pressure, hinge pressure, grouting pressure, grouting volume, tunneling mileage, theoretical excavation volume, actual excavation volume, and shield machine posture after tunneling. Among these, thrust, cutterhead rotational torque, excavation rate, and chamber earth pressure are important factors affecting stratum deformation [60]. Penetration rate, grouting pressure, and cutterhead rotational speed are also considered to be related parameters affecting ground settlement [61,62,63] and therefore are used as input parameters. The operational parameters are shown in Table 4.

### 4.4. Output Parameter

In previous studies, scholars typically used the maximum ground settlement across the entire shield tunnel as the output of prediction models. The maximum ground settlement refers to the peak value of the ground lateral settlement trough caused by tunnel excavation. Typically, the stable value of ground settlement directly above each ring, measured after the shield leaves, is chosen as the maximum ground settlement for that ring [12]. However, for shield tunnel projects with shorter lengths, the number of ground settlement monitoring points outside the tunnel axis is limited, and their distribution is relatively sparse. This makes it challenging to use them as output for prediction models, limiting their practical application in actual engineering projects. Based on the monitoring data from a part of the shield tunnel, this paper uses the maximum settlement value from all ground settlement monitoring points in this part as the output. By including the ground settlement data from all cross-sectional monitoring points, this study aims to maximize the use of limited data for training and assess the predictive capability of machine learning methods under small-sample conditions. The output parameter is shown in Table 5.

## 5. Establishment and Prediction of Maximum Ground Settlement Model

### 5.1. Pre-Processing of Dataset

In this paper, the dataset contains various parameters with different dimensions. In order to prevent the gradient explosion problem during model training, it is necessary to apply minimum–maximum normalization to the dataset before it enters the model. The original data are normalized to the range of [0, 1]. The calculation formula is as follows:

(11) $${m^{\prime }}_{i}=\frac{m_{i}-m_{i\min}}{m_{i\max}-m_{i\min}}$$

In the formula, $m_{i}$ is the i-th parameter, $m_{i\min}$ is the minimum value of the parameter dataset, $m_{i\max}$ is the maximum value of the parameter dataset, and ${m^{\prime }}_{i}$ is the normalized data.

### 5.2. Evaluation Indexes of Model

In this paper, the coefficient of determination (R^2^), mean absolute error (MAE), and root mean square error (RMSE) are used as the evaluation indexes to evaluate the prediction effect of the model. The formulas are as follows:

(12) $$R^{2}=1-\frac{\sum_{i=1}^{n}{\left(y_{i}-{y^{\prime }}_{i}\right)}^{2}}{\sum_{i=1}^{n}{\left(y_{i}-\bar{y}\right)}^{2}}$$

(13) $$MAE=\frac{1}{n}\sum_{i=1}^{n}\left|y_{i}-{y^{\prime }}_{i}\right|$$

(14) $$RMSE=\sqrt{\frac{1}{n}\sum_{i=1}^{n}{\left(y_{i}-{y^{\prime }}_{i}\right)}^{2}}$$

where n is the total number of data samples, $y_{i}$ is the measured ground settlement, ${y^{\prime }}_{i}$ is the settlement value predicted by the model, $\bar{y}$ is the average value of the measured ground settlement, R^2^ is in the range of 0~1 (the larger the value, the better the model prediction effect), MAE represents the mean absolute error between the predicted value and the true value (the smaller the value, the better the model prediction effect), and RMSE represents the root mean square error between the predicted value and the true value, which is more sensitive to large errors, and the smaller the value, the better the model prediction effect.

### 5.3. Selection and Optimization of Model Hyperparameter

This paper adopts particle swarm optimization (PSO) to optimize hyperparameters. The hyperparameters of LSTM and its variant models usually include the number of hidden layers, the number of hidden layer units, the initial learning rate, the optimizer, the dropout value, the number of iterations, the activation function, etc. However, when using the PSO algorithm to find the optimal hyperparameters of LSTM and its variant models, the computational cost increases rapidly as the number of hyperparameters to be optimized increases [1]. According to existing studies [39,64], the number of hidden layers and the number of hidden layer units have a significant impact on the learning ability of the model. Therefore, in order to reduce model calculation time, they are selected as parameters to be optimized, and the parameters are limited to a range of 1 to 3 hidden layers and 10 to 50 hidden layer units. The remaining parameter values [38] are as follows in Table 6:

This study will use the Adam optimizer [65] to accelerate the training process. The training cycle is determined to be 2000 through repeated trials. The training process adopts a step-by-step learning rate. When the number of iterations is less than 1600, the learning rate is 0.01, and when the number of iterations is between 1600 and 2000, the learning rate is 0.001.

This study employs in-model regularization (Dropout) to mitigate the overfitting issue. The Dropout method randomly removes a certain proportion of nodes during each iteration of the neural network, reducing the co-adaptation between neuron nodes and enhancing the model’s generalization capability [25,66].

### 5.4. Model Training and Evaluation Results

This paper collects a total of 126 sets of data, with the first 100 sets used for training and the remaining 26 sets designated as test sets. The division criteria are as follows:

(1) Engineering Temporal Continuity: The shield tunneling process has a strict temporal dependency, and the training and test sets are divided according to the order of shield machine excavation.

(2) Prevention of Data Leakage: Random division could result in future data being included in the training set, so the division is performed in a way that ensures the model evaluation is authentic and reliable.

(3) Test Set Proportion: The number of test sets is based on the “Technical Specifications for Monitoring of Urban Rail Transit Engineering” (GB 50911-2013 [67]) and existing research [68]. The test set proportion (20.6%) meets the standard validation requirements.

**Figure 9:**
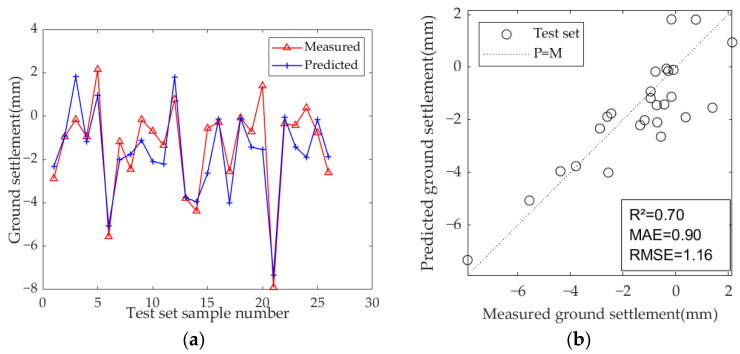
Predicted ground settlement for the test set using the LSTM model: (**a**) training results of test set; (**b**) evaluation results of test set.

**Figure 10:**
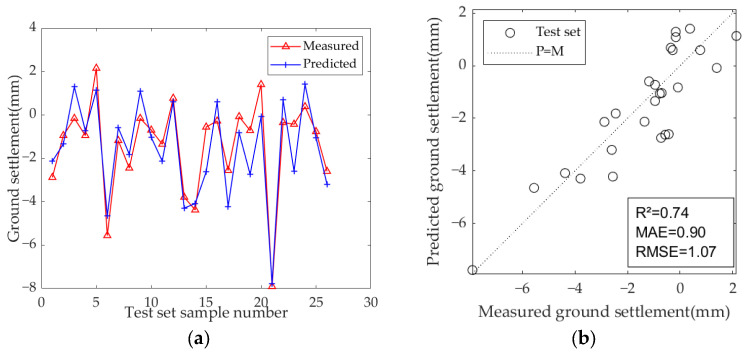
Predicted ground settlement for the test set using the GRU model: (**a**) training results of test set; (**b**) evaluation results of test set.

**Figure 11:**
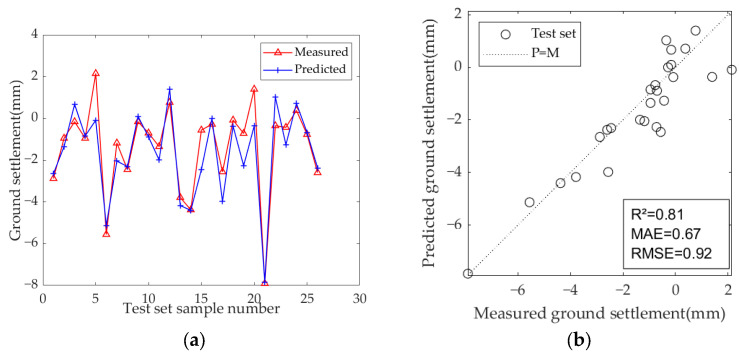
Predicted ground settlement for the test set using the Bi-LSTM model: (**a**) training results of test set; (**b**) evaluation results of test set.

The results of the model test sets are presented in Figure 9, Figure 10 and Figure 11. Table 7 displays the optimal hyperparameters and evaluation metrics of each prediction model.

The results demonstrate that the predicted and measured value curves of LSTM, GRU, and Bi-LSTM models align well, suggesting that these three models perform effectively and can capture the nonlinear relationships between geological parameters, geometric parameters, operational parameters, and ground settlement during the iterative learning process with the training set. All three models can accurately predict the occurrence of large settlement. Among them, the predicted value curve of the Bi-LSTM model in the test set fits the measured curve most closely, with the highest R^2^ of 0.81, and the MAE and RMSE values are the smallest among the three models, which are 0.67 and 0.92, respectively. These results indicate that the Bi-LSTM model has the best prediction performance under complex working conditions and small-sample datasets, followed by the GRU model.

## 6. Correlation Analysis and Model Optimization

### 6.1. Correlation Analysis

This paper employs the Pearson correlation coefficient to analyze the correlations between different input parameters as well as between the input and output parameters, thereby evaluating the linear relationships among the parameters [62,69]. The calculation method is as follows:

(15) $$R=\frac{\sum_{i=1}^{n}\left(x_{i}-{\bar{x}}_{i}\right)\left(x_{j}-{\bar{x}}_{j}\right)}{\sqrt{\sum_{i=1}^{n}{\left(x_{i}-{\bar{x}}_{i}\right)}^{2}}\sqrt{\sum_{i=1}^{n}{\left(x_{j}-{\bar{x}}_{j}\right)}^{2}}}$$

where i and j represent parameters i and j, respectively, ${\bar{x}}_{i}$ and ${\bar{x}}_{j}$ represent the mean values of parameters i and j, respectively, n denotes the total number of data points, and R is the Pearson correlation coefficient, which quantifies the linear relationship between parameters i and j.

The range of the Pearson correlation coefficient is [−1, 1], [−1, 0) indicates negative linear correlation, (0, 1] indicates positive linear correlation, and the larger the absolute value, the stronger the correlation; see Table 8.

**Figure 12:**
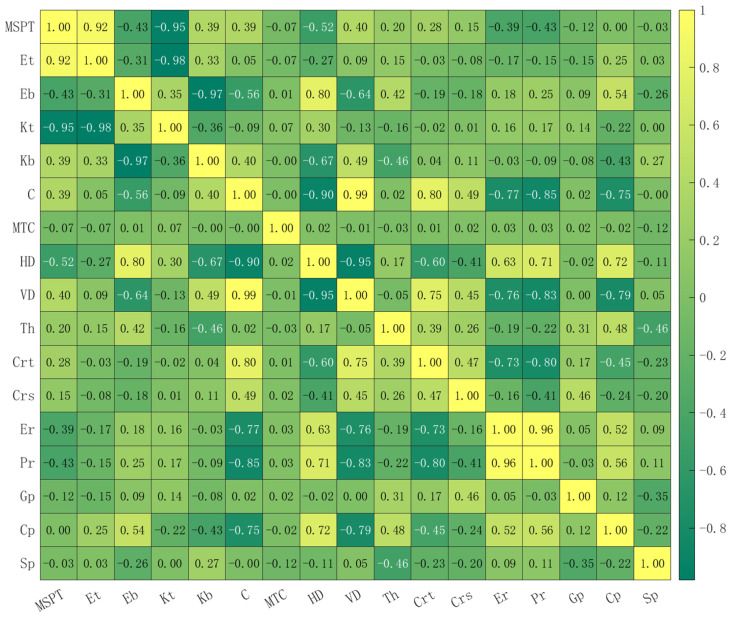
Pearson correlation coefficient between parameters.

The Pearson correlation coefficients of various parameters are shown in Figure 12, and their values are retained to two decimal places. The correlation coefficients between all input parameters and the output parameter ground settlement (Sp) are all less than 0.5, indicating that there is no strong linear relationship between the input parameters and the ground settlement. Only the correlation coefficients between Th and Sp and between Gp and Sp are relatively high, which are −0.46 and −0.35, respectively, indicating that the increase in thrust and grouting pressure reduces the ground settlement, which is consistent with the conclusions of existing studies [57,63]. The Pearson correlation coefficients of Kt, C, and Sp are approximately 0, indicating that these two input parameters have almost no linear relationship with the ground settlement. Among them, MSPT, Eb, MTC, HD, Th, Crt, Crs, Gp, and Cp are negatively correlated with the ground settlement, and Kb, Et, VD, Er, and Pr are positively correlated with the ground settlement.

In the figure, some input parameters exhibit very strong linear correlation. MSPT, Et, and Kt have very strong linear relationships with each other. The Pearson correlation coefficient between MSPT and Et is 0.92, between MSPT and Kt is −0.95, and between Et and Kt is −0.98. In addition, C, HD, and VD also have very strong linear correlations with each other. The linear correlation between C and HD is −0.90, between C and VD is 0.99, and between HD and VD is −0.95. Eb and Kb, as well as Er and Pr, also have very strong linear correlation; the correlation coefficient between Eb and Kb is −0.98, and that between Er and Pr is 0.96.

### 6.2. Model Optimization

**Figure 13:**
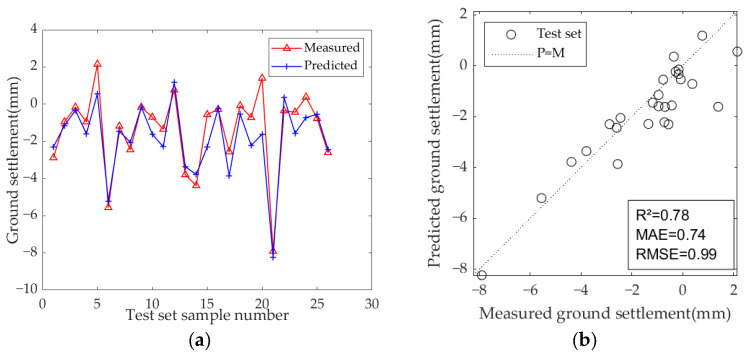
Predicted ground settlement for the test set using the optimized LSTM model: (**a**) training results of test set; (**b**) evaluation results of test set.

**Figure 14:**
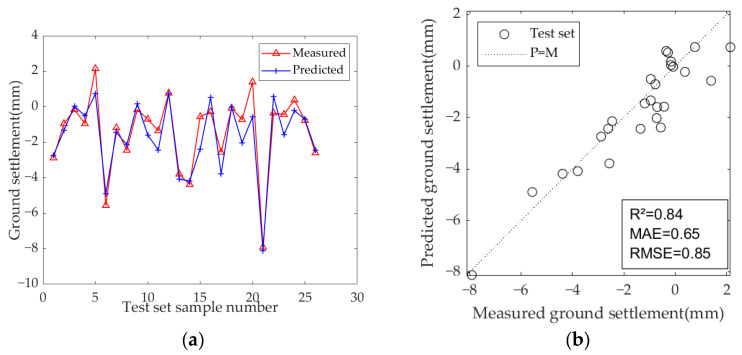
Predicted ground settlement for the test set using the optimized GRU model: (**a**) training results of test set; (**b**) evaluation results of test set.

**Figure 15:**
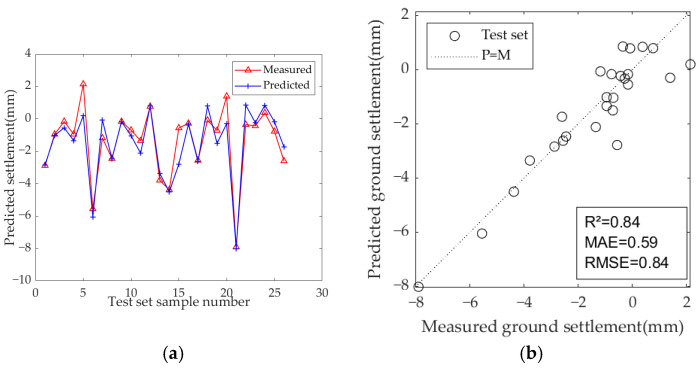
Predicted ground settlement for the test set using the optimized Bi-LSTM model: (**a**) training results of test set; (**b**) evaluation results of test set.

The model input parameters are optimized based on the Pearson correlation coefficient, selecting and retaining one parameter from each pair with a very strong linear correlation. The remaining input parameters after screening are MSPT, Eb, C, Pr, MTC, Th, Crt, Crs, Gp, and Cp, and the optimized model parameters are shown in Table 9. The model training results are presented in Figure 13, Figure 14 and Figure 15, and the optimal hyperparameters and evaluation metrics are summarized in Table 10.

Among the three models, the LSTM and GRU models show significant improvements. The R^2^ of the LSTM model increased from 0.70 to 0.78 (an improvement of +11.4%), indicating enhanced explanatory power for settlement variation. The MAE decreased from 0.90 to 0.74, and the RMSE decreased from 1.16 to 0.99, demonstrating a systematic reduction in prediction bias. The GRU model showed even greater optimization potential, with the R^2^ increasing by 13.5% (from 0.74 to 0.84). Additionally, the MAE decreased from 0.90 to 0.65 (−27.8%) and the RMSE decreased from 1.07 to 0.85 (−20.6%), both reductions being higher than those of the LSTM model. This improvement is attributed to the simplified structure of the GRU and its synergistic optimization with the PSO algorithm. In contrast, the Bi-LSTM model saw a more modest improvement, with R^2^ increasing from 0.81 to 0.84, MAE decreasing from 0.67 to 0.59, and RMSE decreasing from 0.92 to 0.84, which shows that the Bi-LSTM model is more efficient and stable.

### 6.3. Comparison and Analysis

The results demonstrate that after optimizing the selection of input parameters through the Pearson correlation coefficient, the prediction capabilities of the LSTM, GRU, and Bi-LSTM models for ground settlement are all improved. The three models are more accurate in predicting smaller settlements. The reasons for this are as follows:

(1) Eliminating the interference of redundant features. Input parameters with high correlation can lead to redundant information. Removing such redundancy allows the model to focus more on features that are crucial for ground settlement prediction, such as thrust (Th) and grouting pressure (Gp), which can improve the generalization ability of the model.

(2) Reducing the risk of overfitting. The Pearson correlation coefficient reduces the number of input parameters and reduces the risk of model overfitting. Having fewer input features helps reduce noise and improve model performance.

The method of using the Pearson correlation coefficient to screen input parameters is more effective in improving the predictive ability of the LSTM and GRU models, but has less effect on the Bi-LSTM model, which indicates that the prediction performance of the LSTM and GRU models is more sensitive to the selection of input parameters, while the Bi-LSTM model is less sensitive to the interference of highly correlated inputs because it can automatically learn and adapt to the redundant features in the data, making it more stable.

A comparison of the prediction performance of three machine learning models (LSTM, GRU, and Bi-LSTM) optimized using the particle swarm optimization (PSO) algorithm reveals the superior performance of the Bi-LSTM model. The reasons are as follows:

(1) Bidirectional Temporal Dependency Capture: Forward dependency refers to the influence of current construction parameters on the settlement of subsequent monitoring points, while backward dependency indicates that the settlement of subsequent monitoring points affects the adjustment of current construction parameters through soil stress redistribution. The Bi-LSTM model captures this bidirectional coupling mechanism through bidirectional hidden layers (Forward/Backward LSTM), while LSTM/GRU can only capture forward dependencies.

(2) Small Sample Feature Enhancement [70]: When the sample size is limited (*n* = 126), the Bi-LSTM model’s parameter efficiency advantage becomes apparent. The bidirectional structure doubles the gradient update path for each time step in the input sequence, effectively expanding the dataset. Additionally, the fusion of forward and backward hidden states enhances the temporal representation capability per unit parameter.

(3) Synergistic Regularization: The use of the Dropout method complements the bidirectional information redundancy in the Bi-LSTM model, suppressing overfitting while retaining key backward dependency features [71].

### 6.4. Discussion

#### 6.4.1. Limitation

This study validated the reliable predictive performance of the LSTM, GRU, and Bi-LSTM models under complex conditions of shield tunneling undercrossing existing tunnels in composite strata. Although the issue of overfitting was effectively mitigated by introducing the Dropout layer, and the R^2^ difference between the training and test sets was less than 0.1, the small sample size of the dataset (*n* = 126) may still pose the following challenges to the model’s generalization capability:

(1) Insufficient Feature Interaction Learning: A small sample size makes it difficult to fully cover the complex combination space of shield tunneling parameters.

(2) Risk of Local Optima: The optimization process is prone to getting stuck in local extrema with small samples, making the model sensitive to initialization.

(3) Geographical Limitation: The training data are solely from the composite strata in the Hangzhou area, and do not cover other typical geological conditions.

#### 6.4.2. Future Perspectives

Future work will focus on the following two directions:

Enhancing Model Generalization Ability:

(1) Transfer Learning: Pre-train the model using datasets from similar projects in other cities, and reduce the target engineering fine-tuning data requirements through domain adaptation [72].

(2) Data Augmentation: Combine finite element simulations with measured data to generate a mixed dataset that covers a broader range of shield tunneling parameters.

(3) Federated Learning: Build a multi-project collaborative training framework while ensuring data privacy, to enhance the model’s applicability.

(4) Uncertainty Studies: Try to use different methods such as Monte Carlo (MC) sampling variance and prediction entropy to measure the uncertainty of ground settlement monitoring values [73], and study the impact of this uncertainty on the model prediction performance.

Real-time Settlement Monitoring and Early Warning System:

(1) Multi-scale Monitoring System Construction: While electronic leveling instruments offer sub-millimeter sensitivity in vertical displacement monitoring, their single-point measurement characteristics may lead to the omission of local settlement features. Future research could integrate the wide-area coverage advantages of InSAR to build a multi-scale monitoring system [74].

(2) Graded Early Warning Mechanism: Based on relevant standards (such as “Urban Rail Transit Engineering Measurement Code” (GB/T 50308-2017 [75]) and “Urban Rail Transit Engineering Monitoring Technical Code” (GB 50911-2013)), as well as the actual project situation, a grading early warning system for ground settlement (safety, low risk, warning, danger) should be established, along with corresponding emergency response plans for each level.

(3) Model Ensemble Prediction: Integrate the PSO-Bi-LSTM model into the monitoring system, and use the graded early warning mechanism to determine whether the predicted values fall within the warning or danger range for proactive responses.

## 7. Conclusions

This paper uses the ground settlement caused by the construction of a new shield tunnel in Hangzhou, Zhejiang Province, China, as a case study. Faced with the complex working conditions of poor foundation composite strata and the undercrossing of an existing tunnel, the ground settlement was predicted using machine learning methods. The following conclusions can be drawn:

(1) Comprehensive Ground Settlement Monitoring Layout: The left line of the newly constructed shield tunnel adopts three different ground settlement monitoring layout methods, which allow for more comprehensive monitoring of ground settlement. The obtained data effectively analyze the longitudinal ground settlement curve, and machine learning methods can predict the maximum ground settlement at the monitoring points. This monitoring layout is both scientifically sound and cost-effective.
(2) Observed Ground Settlement Characteristics: According to the measured data of cross-sectional ground settlement, before the excavation surface reaches the monitoring section, the ground experiences slight settlement or uplift, with a settlement range of about ±2 mm. After the excavation surface reaches the monitoring section, ground settlement increases. The settlement tends to stabilize when the excavation surface is 40–60 m away from the monitoring section, and the normalized average final ground settlement value of the cross-section aligns well with the curve derived from the Peck empirical formula (with i = 6.5).
(3) Machine Learning Model Performance: Using machine learning methods, it was verified that the LSTM, GRU, and Bi-LSTM models, after hyperparameter optimization through particle swarm optimization (PSO), could effectively predict ground settlement using small sample data under the complex working conditions of crossing composite strata and undercrossing an existing tunnel. The model prediction abilities were ranked as follows: Bi-LSTM > GRU > LSTM.
(4) Correlation Analysis of Model Parameters and model optimization: A Pearson correlation coefficient analysis was performed on the model parameters, revealing that none of the input parameters had a strong linear relationship with the output parameter, ground settlement. Only thrust and grouting pressure showed a relatively high linear correlation with ground settlement. Additionally, there were extremely strong linear relationships between some input parameters. Parameters with excessively high linear correlations were removed, and those with low linear correlation were retained. The ground settlement was then predicted again using machine learning methods. The results showed that after optimizing the number of input parameters through Pearson correlation coefficient analysis, the prediction capabilities of all three models improved. Among them, LSTM and GRU showed significant improvement, while Bi-LSTM showed a slight improvement. This indicates that LSTM and GRU models are more dependent on the selection of input parameters, while the Bi-LSTM model is less sensitive to input parameter selection, can handle highly correlated inputs, and is more stable.

## Figures and Tables

**Table 1:** The physical and mechanical parameters of the soil layers passed through by the 0~250 rings of the left line of the shield tunnel.

Soil Layer Number	Name of Soil Layer	Unit Weights (kN·m^−3^)	Void Ratio	Elastic Modulus (Mpa)	Standard Penetration Test Blow Count	Cohesion (kPa)	Internal Friction Angle (°)	Static Lateral Pressure Coefficient (m·s^−1^)
②_4_	sandy silt	19.10	0.80	6.24	12.00	5.00	26.00	0.52
③_5_	silty sand mixed with sandy silt	19.30	0.73	20.7	16.10	5.00	32.00	0.37
③_7_	sandy silt	19.20	0.82	6.72	9.70	7.00	24.00	0.52
⑥_2_	mucky silty clay mixed with silty clay	18.20	1.03	3.12	(3.60)	12.00	16.00	0.58
⑦_2_	silty clay	19.70	0.74	12.96	17.50	28	18.00	0.47

**Table 2:** Geological parameters of the prediction model.

**Category**	**Parameter**	**Abbreviation**	**Unit**	**Input/Output**
Geological parameters	Modified standard penetration test blow count	MSPT		Input
	Elastic modulus of top soil layer	Et	MPa	Input
	Elastic modulus of bottom soil layer	Eb	MPa	Input
	Static lateral pressure coefficient of the top soil layer	Kt	m·s^−1^	Input
	Static lateral pressure coefficient of the bottom soil layer	Kb	m·s^−1^	Input

**Table 3:** Geometric parameters of the prediction model.

Category	Parameter	Abbreviation	Unit	Input/Output
Geometric parameters	Cover depth	C	m	Input
	Distance between monitoring point and central axis	MTC	m	Input
	Horizontal distance	HD	m	Input
	Vertical dimension	VD	m	Input

**Table 4:** Operational parameters of the prediction model.

Category	Parameter	Abbreviation	Unit	Input/Output
Operational parameters	Thrust	Th	MN	Input
	Cutterhead rotational torque	Crt	KN × m	Input
	Cutterhead rotational speed	Crs	r/min	Input
	Excavation rate	Er	mm/min	Input
	Penetration rate	Pr	mm/min	Input
	Grouting pressure	Gp	bar	Input
	Chamber earth pressure	Cp	bar	Input

**Table 5:** Output parameter of the prediction model.

Category	Parameter	Abbreviation	Unit	Input/Output
Settlement	Maximum ground settlement of monitoring point	Sp	mm	Output

**Table 6:** Selection of model hyperparameters.

Hyperparameters	Initial Learning Rate	Optimizer	Iterations	Activation Function
value	0.01	Adam	2000	ReLU

**Table 7:** The optimal hyperparameters and evaluation metrics of each model.

Model	Optimal Hyperparameters	R^2^	MAE	RMSE
LSTM	number of hidden layers: 1 number of hidden layer units: 28	0.70	0.90	1.16
GRU	number of hidden layers: 1 number of hidden layer units: 18	0.74	0.90	1.07
Bi-LSTM	number of hidden layers: 2 number of hidden layer units: 24	0.81	0.67	0.92

**Table 8:** The absolute value of the Pearson correlation coefficient corresponds to the degree of linear correlation.

|R|	Linear Correlation
0	no linearity
(0, 0.2]	very weak correlation
(0.2, 0.4]	weak correlation
(0.4, 0.6]	moderate correlation
(0.6, 0.8]	strong correlation
(0.8, 1]	very strong correlation

**Table 9:** The optimized model parameters.

Category	Parameter	Abbreviation	Unit	Input/Output
Geological parameters	Modified standard penetration test blow count	MSPT		Input
	Elastic modulus of bottom soil layer	Eb	MPa	Input
Geometric parameters	Cover depth	C	m	Input
	Distance between monitoring point and central axis	MTC	m	Input
Operational parameters	Thrust	Th	MN	Input
	Cutterhead rotational torque	Crt	KN × m	Input
	Cutterhead rotational speed	Crs	r/min	Input
	Penetration rate	Pr	mm/min	Input
	Grouting pressure	Gp	bar	Input
	Chamber earth pressure	Cp	bar	Input
Settlement	Maximum ground settlement of monitoring point	Sp	mm	Output

**Table 10:** The optimal hyperparameters and evaluation metrics of each optimized model.

Model	Optimal Hyperparameters	R^2^	MAE	RMSE
Optimized LSTM	number of hidden layers: 1 number of hidden layer units: 25	0.78	0.74	0.99
Optimized GRU	number of hidden layers: 3 number of hidden layer units: 16	0.84	0.65	0.85
Optimized Bi-LSTM	number of hidden layers: 2 number of hidden layer units: 13	0.84	0.59	0.84

## Data Availability

Data available on request due to restrictions.
